# Description of two new species of the *Exocelina broschii*-group from Papua New Guinea, with revision and key to all representatives of this species group (Coleoptera, Dytiscidae, Copelatinae)

**DOI:** 10.3897/zookeys.577.7254

**Published:** 2016-04-05

**Authors:** Helena Shaverdo, Katayo Sagata, Michael Balke

**Affiliations:** 1 Naturhistorisches Museum, Burgring 7, 1010 Vienna, Austria; 2 Papua New Guinea Institute of Biological Research (PNGIBR), Goroka, Papua New Guinea; 3 SNSB-Zoologische Staatssammlung München, Münchhausenstraße 21, D-81247 Munich, Germany and GeoBioCenter, Ludwig-Maximilians-University, Munich, Germany

**Keywords:** Exocelina broschii-group, Copelatinae, Dytiscidae, new species, Papua New Guinea

## Abstract

Two new species of *Exocelina* Broun, 1886 from Papua New Guinea are described herein: *Exocelina mondmillensis* **sp. n.** and *Exocelina pseudomarinae* **sp. n.** They are placed into the *Exocelina broschii*-group based on the shovel/fork-like ventral sclerites of their median lobe. While the former has rather distinct combination of the morphological characters (inconspicuous dorsal punctation, thin apex of the median lobe and ventral sclerite of the median lobe with two tips of different length), the latter is very similar to already described species *Exocelina marinae* (Shaverdo, Sagata & Balke, 2005). All described species of the group are revised and a key to their identification is provided. Important diagnostic characters (habitus, color, protarsomeres 4–5, median lobes, and parameres) are illustrated. Data on the distribution of all species of the group are given showing that its representatives occur only in Papua New Guinea and most of them are widely distributed in it central part.

## Introduction

This paper continues our previous studies on the New Guinea species of the genus *Exocelina* Broun, 1886 (Balke 1998, 1999; Shaverdo and Balke 2014; Shaverdo et al. 2005, 2012, 2013, 2014, 2016, submitted) and deals with one of the five species groups of New Guinea *Exocelina*, the *Exocelina broschii*-group. This group was introduced by Shaverdo et al. (2005) for three species from Papua New Guinea (*Exocelina broschii* (Balke, 1998), *Exocelina hintelmannae* (Shaverdo, Sagata & Balke, 2005) and *Exocelina marinae* (Shaverdo, Sagata & Balke, 2005)) and defined by the following apomorphy: shovel/fork-like ventral sclerite of the median lobe. Monophyly of the group was also supported by the phylogenetic analysis, based on molecular data (Toussaint et al. 2014).

Here, we provide a detailed diagnosis of the *Exocelina broschii*-group, describe two new species, review the known species providing new faunistic data, and present a key to the species and map of their distribution.

We provided electronic resources for the species treated here in the form of species pages, which were automatically created by ZooKeys on the species-id.net portal with the publication of this article. This wiki engine based site provides for example high resolution art work and can be improved interactively should new data become available. By providing these resources, we hope to help creating a more user-friendly, sustainable taxonomy as suggested by Riedel et al. (2013).

Including the results of this work, 91 *Exocelina* species are described from New Guinea and 144 worldwide.

## Material and methods

The present work is based on the material from the following collections:

BMNH
 The Natural History Museum, London, UK 


NARI
 Papua New Guinea National Insect Collection, Port Moresby, PNG 


NHMW
Naturhistorisches Museum Wien, Vienna, Austria 


ZSM
 Zoologische Staatsammlung München, Munich, Germany 


All specimen data are quoted as they appear on the labels attached to the specimens. Label text is cited using quotation marks. Comments in square brackets are ours. We extracted DNA and obtained DNA sequence data for some of the species/specimens, marked with individual DNA extraction numbers (e.g., “264 DNA M. Balke”). All types of the herein described species are provided with red labels. The female specimens, identification of which is difficult or sometimes impossible, were included in the type series only when collected with males of respective species and did not differ morphologically from them. If two or more morphologically similar species were collected together (i.e., males found together), their females were not included in the types series of the respective species but were instead mentioned under additional material. Species descriptions are based on the whole type series.

Some of the species treated herein are very similar to each other and, based on low overall genetic divergence, most likely also very recent (Toussaint et al. 2014). We have used constant morphological difference based on examined series as an indicator of interrupted gene flow and as an operational criterion to delineate biological species, but suggest that extensive population genetic work using genomic data might reveal many additional lineages that represent putative species in a highly structured geographic and geological setting.

Measurements were taken with a Wild M10 stereomicroscope choosing the smallest and the largest specimens within and among the populations. The following abbreviations were used: TL (total body length), TL-H (total body length without head), MW (maximum body width), and hw (handwritten). The number of ventral setae on the male protarsomere 5 is given for only one specimen of each species, which was mounted on a glass slide (see below) for drawing. This character was found not very useful for the species identification since it is possible to make a general statement of the setation pattern (short/long, dense/sparse) but not to count them with certainty at the magnification of normal dissecting scopes. The potential phylogenetic information content of this character will be studied in a further work.

Drawings were made with the aid of a camera lucida attached to a Leica DM 2500 microscope. For detailed study and drawing, protarsi, and genitalia were removed and mounted on glass slides with DMHF (dimethyl hydantoin formaldehyde) as temporary preparations. The drawings were scanned and edited, using the software Adobe Illustrator CS5.1. Arrangement of the figures follows the species descriptions.

The terminology to denote the orientation of the genitalia (ventral for median lobe and dorsal and external for paramere) follows Miller and Nilsson (2003). Left and right lobes of the ventral sclerite of the median lobe are indicated according figure view, not their original orientation. The terminology on the structure of the prosternum follows Larson et al. (2000). Administrative divisions of Papua New Guinea follow information from Wikipedia (2016).

## Diagnosis of the *Exocelina broschii*-group sensu Shaverdo et al. (2005)

The representatives of the *Exocelina broschii*-group share the following diagnostic characters:

- beetles small or middle-sized (TL-H 3.2–4.15 mm);
- habitus oblong-oval (broadest approximately at elytral middle), with rounded pronotal and elytral sides, body outline continuous;
- pronotum short, trapezoidal, with posterior angles not drawn backwards;
- coloration brown to piceous, mainly uniform, sometimes with paler head and pronotum and darker elytra;
- microreticulation and punctation of dorsal surface very fine to strongly impressed, so that beetles shiny to matt dorsally;
- metacoxae and abdominal ventrites 1–5 (and 6 in males) with thin, almost longitudinal striae/strioles;
- pronotum and elytra without striae or strioles;
- pronotum with lateral bead;
- male antennomeres not modified, antennomere 2 larger than antennomere 3;
- male protarsomeres 1–3 not expanded laterally;
- male protarsomere 4 cylindrical, narrow, with large anterolateral hook;
- male protarsomere 5 not modified: long and narrow, without expansion and concavity, ventrally with two sparse rows of relatively short setae;
- median lobe of aedeagus with continuous outline in ventral and lateral view;
- ventral sclerite of median lobe not deeply divided in the middle, apically forming a shovel/fork-like structure with two apices;
- apical part of median lobe with numerous setae;
- paramere without notch on dorsal side;
- paramere with long setae occupying whole dorsal side.

## Checklist and distribution of the species of the *Exocelina broschii*-group

Representatives of this species group are recorded only from Papua New Guinea (PNG).

**Table:** Representatives of this species group are recorded only from Papua New Guinea (PNG).

1.	*Exocelina broschii* (Balke, 1998)	PNG Madang, Eastern Highlands
2.	*Exocelina hintelmannae* (Shaverdo, Sagata & Balke, 2005)	PNG: Simbu, Eastern Highlands, Gulf
3.	*Exocelina marinae* (Shaverdo, Sagata & Balke, 2005)	PNG: Sandaun, Hela
4.	*Exocelina mondmillensis* sp. n.	PNG: Western Highlands, Enga, Madang
5.	*Exocelina pseudomarinae* sp. n.	PNG: Hela

## Species descriptions

### 1. Exocelina broschii

Taxon classificationAnimaliaColeopteraDytiscidae

(Balke, 1998)

Figs 1–3 , 8 , 9A–E

Copelatus (Papuadytes) broschii Balke, 1998: 327; Nilsson 2001: 76 (catalogue).
Papuadytes broschii (Balke, 1998): Shaverdo et al. 2005: 270, 271 (notes, illustration).
Papuadytes broschii (Balke, 1998): Nilsson and Fery 2006: 56 (addition to catalogue).
Exocelina broschii (Balke, 1998): Nilsson 2007: 33 (comb. n.).
Exocelina undescribed sp. MB1520: Toussaint et al. 2014: Supplementary figs 1–4, tab. 2.

#### Type locality.

Papua New Guinea: Madang Province, Finisterre Range, Moro, approximately 5°42'47.6"S; 146°03'40.1"E.

#### Type material studied.

*Holotype*: male “Stn. No. 82”, “NEW GUINEA: Madang Dist., Finisterre Mts. Moro.C.5550ft. 30.x.-15.xi.1964.”, “M.E. Bacchus. B.M. 1965-120”, “Holotypus” [red], “Copelatus broschii sp.n. Balke des. 1997” (BMNH). *Paratypes*: 4 males, 1 female with the same labels as the holotype, except for “Paratypus Copelatus broschii sp.n. Balke des. 1997” (BMNH, NHMW, ZSM).

#### Additional material.

**Madang**: 1 male “Stn. No. 82”, “NEW GUINEA: Madang Dist., Finisterre Mts. Moro.C.5550ft. 30.x.-15.xi.1964.”, “M.E. Bacchus. B.M. 1965-120”, “Paratypus Copelatus broschii sp.n. Balke des. 1997” (BMNH) – although this specimen is with the paratype label, it is not included in the type material of the original description in Balke (1998). 17 males, 8 females “Papua New Guinea: Madang, Adalbert Mts., Sewan - Keki, 700m, 4.v.2006, 04.42.215S 145.25.154E, Balke & Manaono (PNG 51)” (NARI, NHMW, ZSM). 7 males “Papua New Guinea: Madang, Adalbert Mts., Keki, 850m, 4.v.2006, nr 04.42.300S 145.25.089E, Balke & Manaono (PNG 52)”, one male with an additional green label “DNA M.Balke 1300” (NHMW, ZSM). 34 males, 35 females “Papua New Guinea: Madang, Adalbert Mts., below Keki, 790m, 5.v.2006, 04.42.300S 145.25.089E, Balke & Manaono (PNG 53)” (NARI, NHMW, ZSM). 14 males, 13 females “Papua New Guinea: Madang, Adalbert Mts., creek nr Keki, 790m, 28.xi.2006, 04.42.300S 145.25.089E, Binatang Boys leg. (PNG 53a)” (NHMW, ZSM). 4 males, 5 females “Papua New Guinea: Madang, Keki, Adalbert Mts., 400m, 29.xi.2006, 04.43.058S 145.24.437E, Binatang Boys, (PNG 119)” (NHMW, ZSM). 7 males, 6 females “Papua New Guinea: Madang, Keki-Sewan, Adalbert Mts., 700m, 30.xi.2006, nr 04.41.802S 145.25.460E Binatang Boys (PNG 120)” (NHMW, ZSM). 1 male “Papua New Guinea: Madang, Simbai area, 1800-2400m, 8.iii.2007, 05.12.693S 144.35.521E, Kinibel (PNG 151)” (ZSM). 24 males “Papua New Guinea: Madang, Simbai area, 1200m, 10.iii.2007, 05.13.389S 144.37.285E, Kinibel (PNG 152)” (NARI, NHMW, ZSM). 39 males “Papua New Guinea: Madang, Simbai area, 1200m, 11.iii.2007, 05.13.333S 144.37.611E, Kinibel (PNG 153)” (NARI, NHMW, ZSM). 10 males, 2 females “Papua New Guinea: Madang, Simbai - Mombeen, 1100m, 11.iii.2007, 05.12.876S 144.41.759E, Kinibel (PNG 154)” (NHMW, ZSM). **Eastern Highlands**: 4 males, 6 females “Papua New Guinea: Eastern Highlands, Bena Bridge, 1400m, 8.xii.2007, 06.10.781S 145.26.034E, Balke & Sagata (PNG 164)” (ZSM). 2 males “Papua New Guinea: Eastern Highlands, Akameku - Brahmin, Bismarck Range, 700m, 24.xi.2006, 05.52.754S 145.23.209E, Balke & Kinibel (PNG 109)”, one of them with an additional green label “DNA M.Balke 1520” (ZSM). 1 male, 2 females “Papua New Guinea: Eastern Highlands, Akameku - Brahmin, Bismarck Range, 1200m, 24.xi.2006, nr 05.52.754S 145.23.209E, Balke & Kinibel (PNG 110)”, one female with an additional green label “DNA M.Balke 1522” (ZSM). 8 males “Papua New Guinea: Eastern Highlands, Akameku - Brahmin, Bismarck Range, 800m, 24.xi.2006, 05.50.021S 145.24.664E, Balke & Kinibel (PNG 112)” (NHMW, ZSM). 18 males “Papua New Guinea: Eastern Highlands, Akameku - Brahmin, Bismarck Range, 750m, 25.xi.2006, 05.49.892S 145.24.491E, Balke & Kinibel (PNG 113)” (NARI, NHMW, ZSM). 3 males “Papua New Guinea: Madang, Akameku - Brahmin, Bismarck Range, 750m, 25.xi.2006, nr 05.49.307S 145.24.389E, Balke & Kinibel (PNG 114)” (NHMW, ZSM).

**Females of doubtful identity. Eastern Highlands**: 12 females “Papua New Guinea: Eastern Highlands, Akameku - Brahmin, Bismarck Range, 700m, 24.xi.2006, 05.52.754S 145.23.209E, Balke & Kinibel (PNG 109)” (ZSM). 24 females “Papua New Guinea: Eastern Highlands, Akameku - Brahmin, Bismarck Range, 800m, 24.xi.2006, 05.50.021S 145.24.664E, Balke & Kinibel (PNG 112)” (NARI, NHMW, ZSM). 15 females “Papua New Guinea: Eastern Highlands, Akameku - Brahmin, Bismarck Range, 750m, 25.xi.2006, 05.49.892S 145.24.491E, Balke & Kinibel (PNG 113)” (NHMW, ZSM). 25 females “Papua New Guinea: Madang, Akameku - Brahmin, Bismarck Range, 750m, 25.xi.2006, nr 05.49.307S 145.24.389E, Balke & Kinibel (PNG 114)” (NARI, NHMW, ZSM). These females are a mixture of two species: *Exocelina broschii* and *Exocelina damantiensis* (Balke, 1998). 19 males “Papua New Guinea: Madang, Simbai area, 1200m, 10.iii.2007, 05.13.389S 144.37.285E, Kinibel (PNG 152)” (NHMW, ZSM). These females are a mixture of two species: *Exocelina broschii* and Exocelina ? damantiensis (Balke, 1998). 53 females “Papua New Guinea: Madang, Simbai area, 1200m, 11.iii.2007, 05.13.333S 144.37.611E, Kinibel (PNG 153)” (NARI, NHMW, ZSM). These females are a mixture of three species: *Exocelina broschii*, *Exocelina simbaiarea* Shaverdo & Balke, 2014, and Exocelina ? damantiensis (Balke, 1998).

#### Additions to the description

(original description in Balke 1998, p. 327). *Size and shape*: Beetles small to medium-sized (TL-H 3.2–4.0 mm, TL 3.7–4.4 mm, MW 1.75–2.15 mm; holotype: TL-H 3.55 mm, TL 3.9 mm, MW 1.9 mm). *Male*: Protarsomere 4 with large, thick, strongly curved anterolateral hook-like seta. Protarsomere 5 ventrally with anterior row of 14 and posterior row of 5 short setae (Fig. 8A). Abdominal ventrite 6 with 3–6 lateral striae on each side. Median lobe with slightly curved apex in lateral view and more or less rounded in ventral view. Its ventral sclerite with almost equal, short apical lobes: left lobe broad or narrow (sometimes with broken apex, e.g., Fig. 9A) and right lobe short, relatively broad (Fig. 8B, C).

**Figures 1–3.**
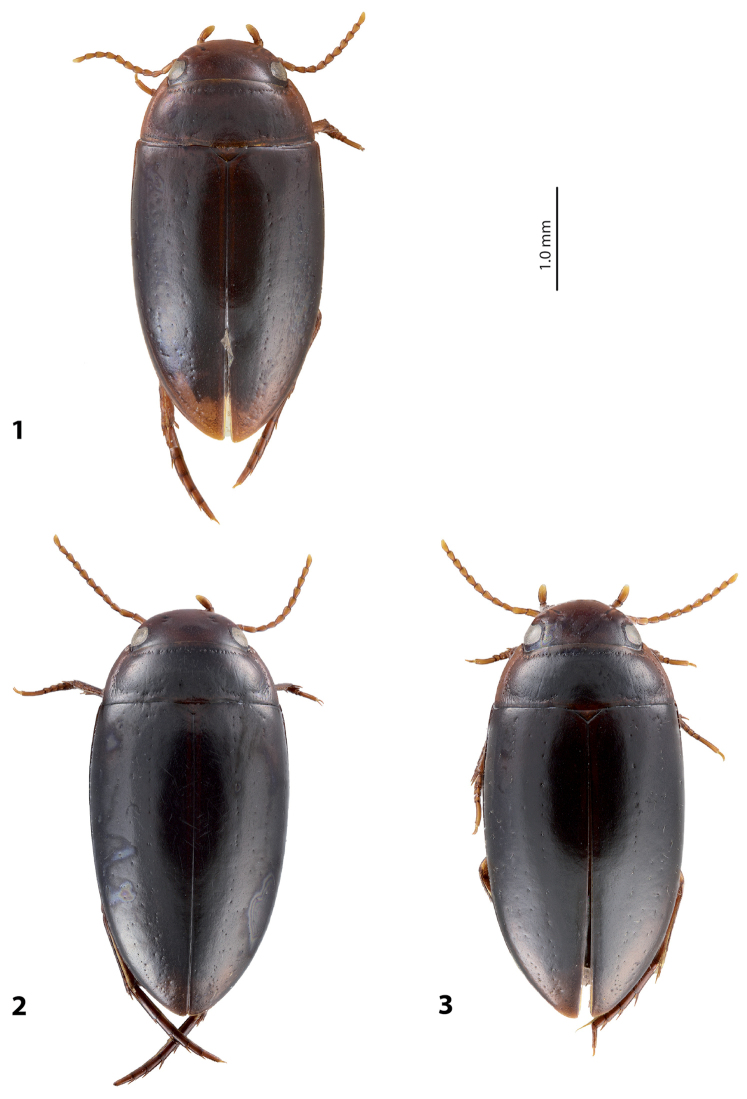
Habitus and coloration of *Exocelina broschii* (Balke, 1998). **1** holotype **2** Madang, Simbai area, PNG 152, specimen with finer dorsal punctation **3** Madang, Simbai area, PNG 152, specimen with coarser dorsal punctation.

**Figures 4–7.**
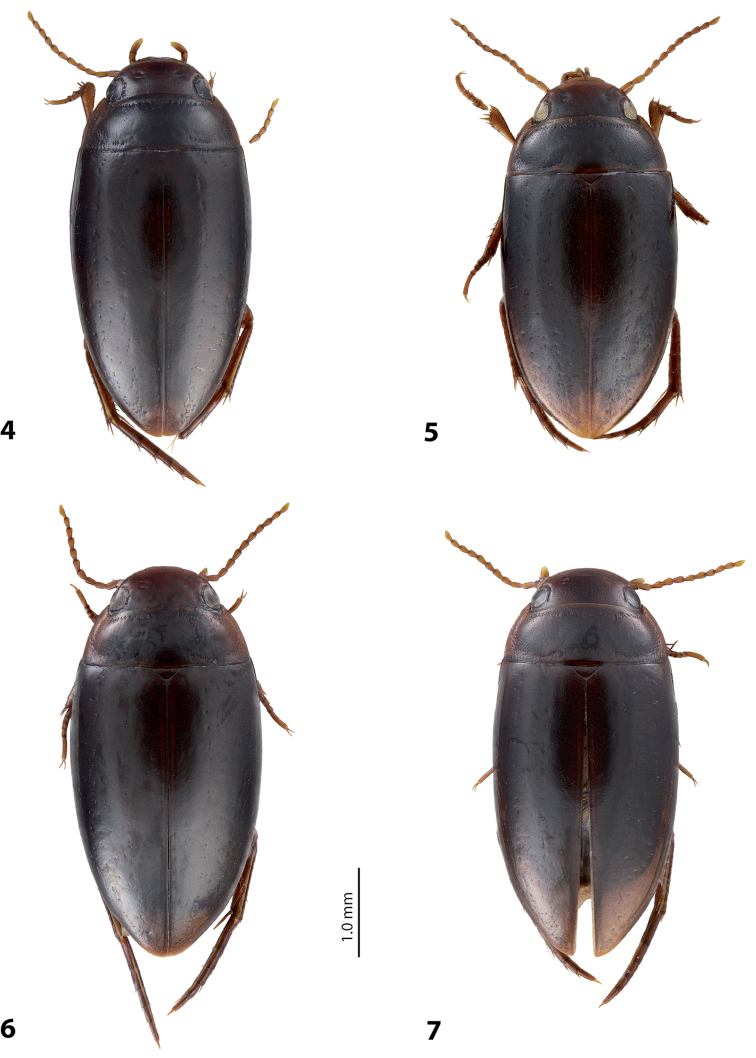
Habitus and coloration of the holotypes. **4** *Exocelina hintelmannae* (Shaverdo, Sagata & Balke, 2005) **5** *Exocelina marinae* (Shaverdo, Sagata & Balke, 2005) **6** *Exocelina mondmillensis* sp. n. **7** *Exocelina pseudomarinae* sp. n.

**Figure 8.**
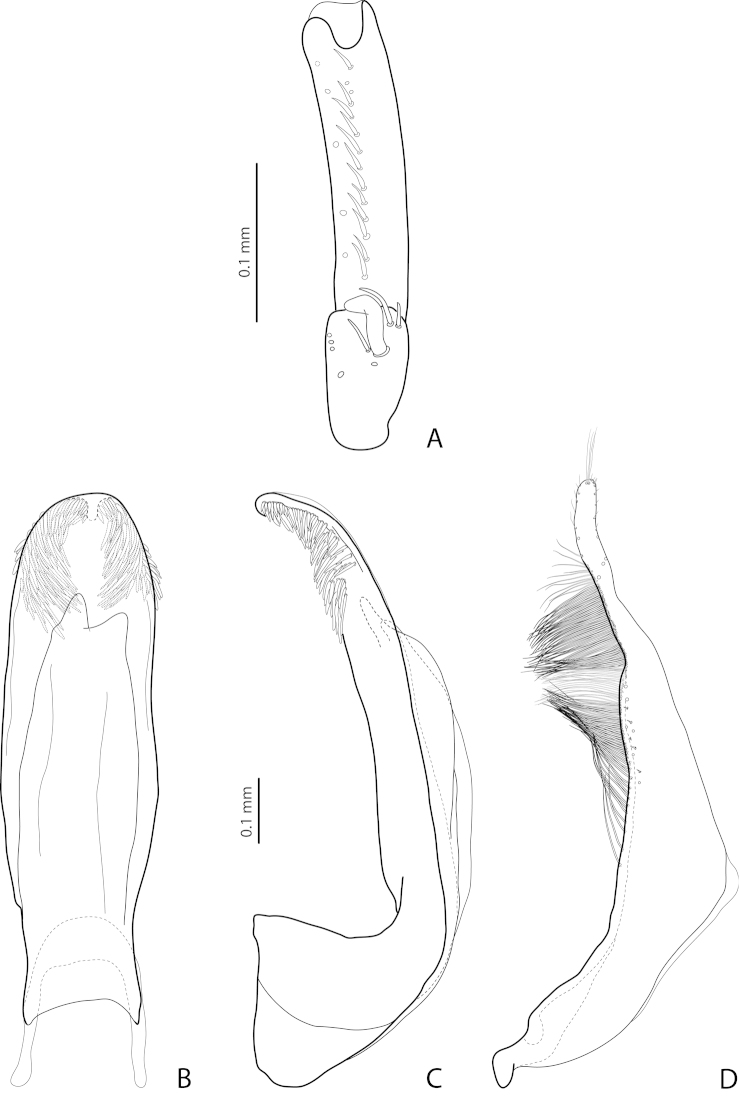
*Exocelina broschii* (Balke, 1998). **A** male protarsomeres 4–5 in ventral view (Madang, Simbai area, PNG 153) **B** median lobe in ventral view (paratype) **C** median lobe in lateral view (paratype) **D** paramere in external view (Madang, Simbai area, PNG 153).

**Figure 9.**
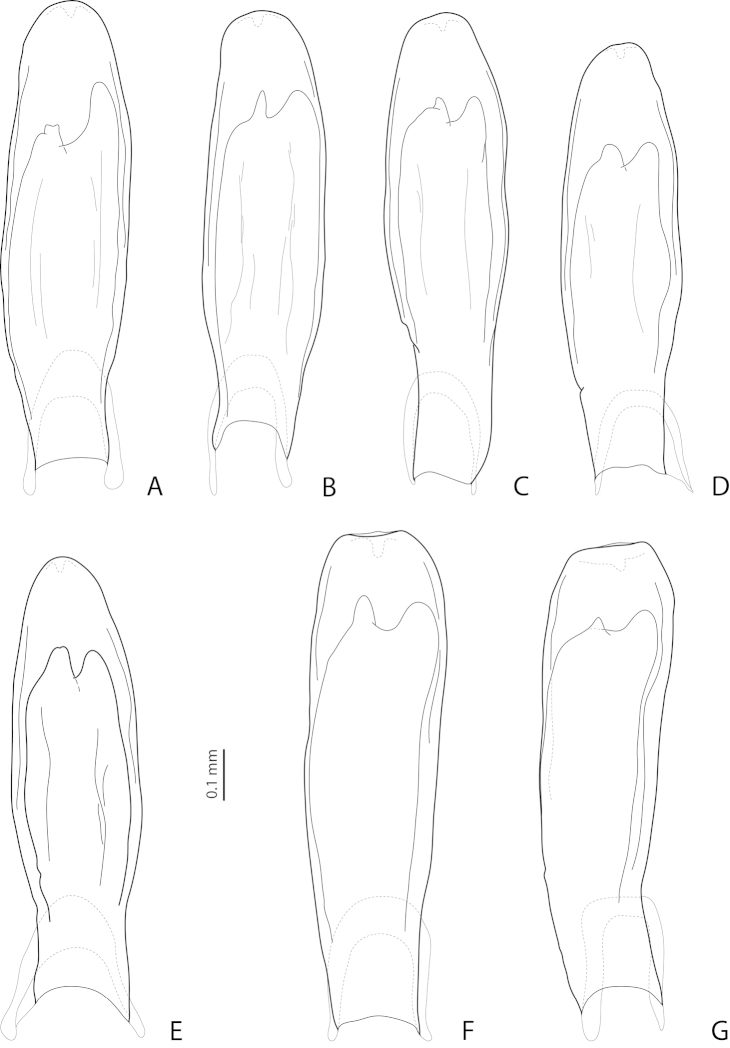
Lateral view of median lobe of **(A–E)** *Exocelina broschii* (Balke, 1998) and **(F, G)** *Exocelina hintelmannae* (Shaverdo, Sagata & Balke, 2005). **A, B** Madang, Simbai area, PNG 153 **C** Madang, Adalbert Mts., Keki, PNG 52 **D** Madang, Adalbert Mts., below Keki, PNG 53 **E** Eastern Highlands, Bena Bridge, PNG 164 **F, G** Simbu/ Eastern Highlands, Crater Mountain, Sera-Herowana, Hulene River, PNG 017. Setae not shown.

**Figure 10.**
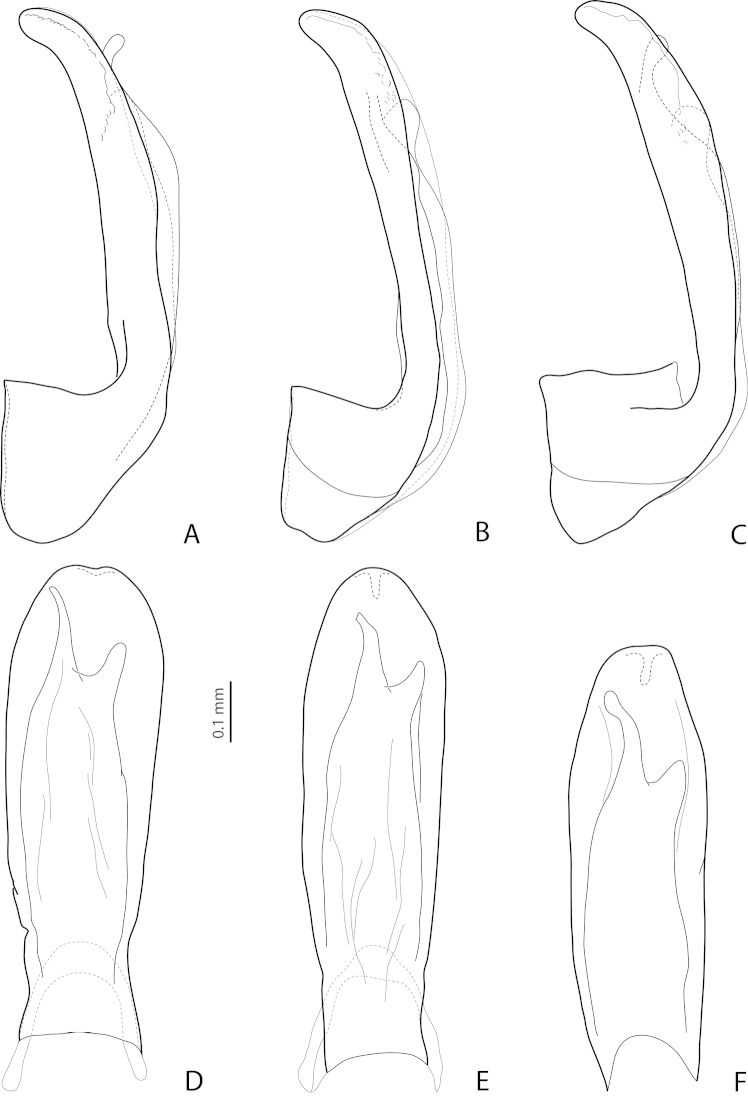
*Exocelina marinae* (Shaverdo, Sagata & Balke, 2005), lateral **(A–C)** and ventral **(D–F)** views of median lobe. **A, D** Sandaun, Mianmin, PNG 196 **B, E** Sandaun, Mianmin area, PNG 234 **C, F** Southern Highlands, Tari to Koroba, PNG 65. Setae not shown.

*Variability*: Beetles vary in size, kind of dorsal punctation (Figs 1–3), and shape of ventral sclerite of the median lobe (Fig. 9A–E). Dorsal punctation in great majority of the specimens is fine (Fig. 3), but some specimens have slightly coarser punctation, and very few (the types and one specimen from Simbai area, Madang, see Figs 1, 2) have distinct punctation, similar to that of *Exocelina pseudomarinae* sp. n. There are populations (e.g., from Simbai area, Madang, and Akameku-Brahmin, Eastern Highlands) with the left lobe of the ventral sclerite of the median lobe very narrow (Fig. 9A, B). However, this character is not very stable even in one population. Taking into consideration the other different shapes of the ventral sclerite observed (Figs 8B, 9C–E) as well as the fact that it is differently sclerotized in different specimens and because of that is variable in shape, we treated all the material as *Exocelina broschii*. Such variability in shape of the ventral sclerite of the median lobe is also characteristic for other species of this group (Figs 9, 10, 12).

**Figure 11.**
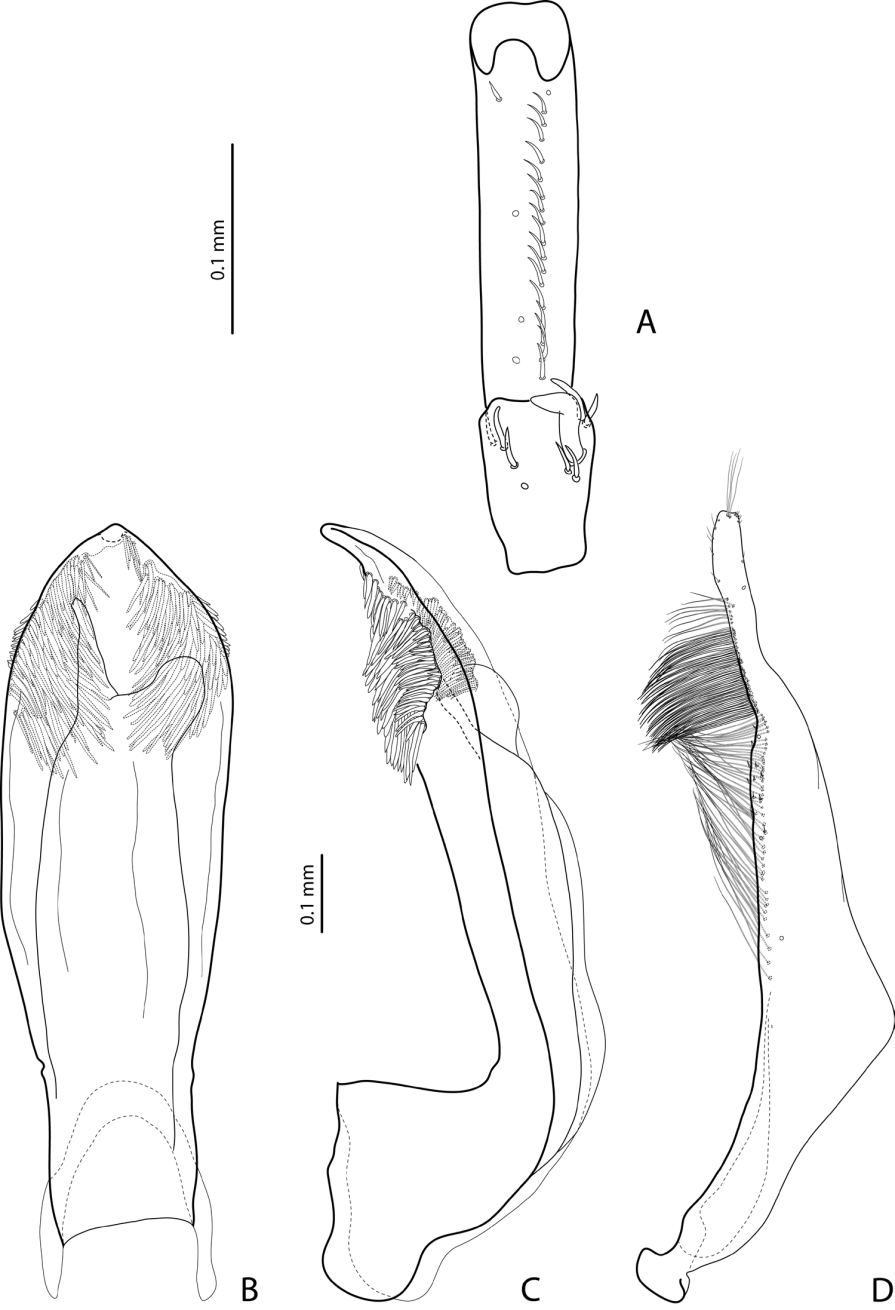
*Exocelina mondmillensis* sp. n., Western Highlands, Mondmill, PNG 77 **A** male protarsomeres 4–5 in ventral view **B** median lobe in ventral view **C** median lobe in lateral view **D** paramere in external view.

**Figure 12.**
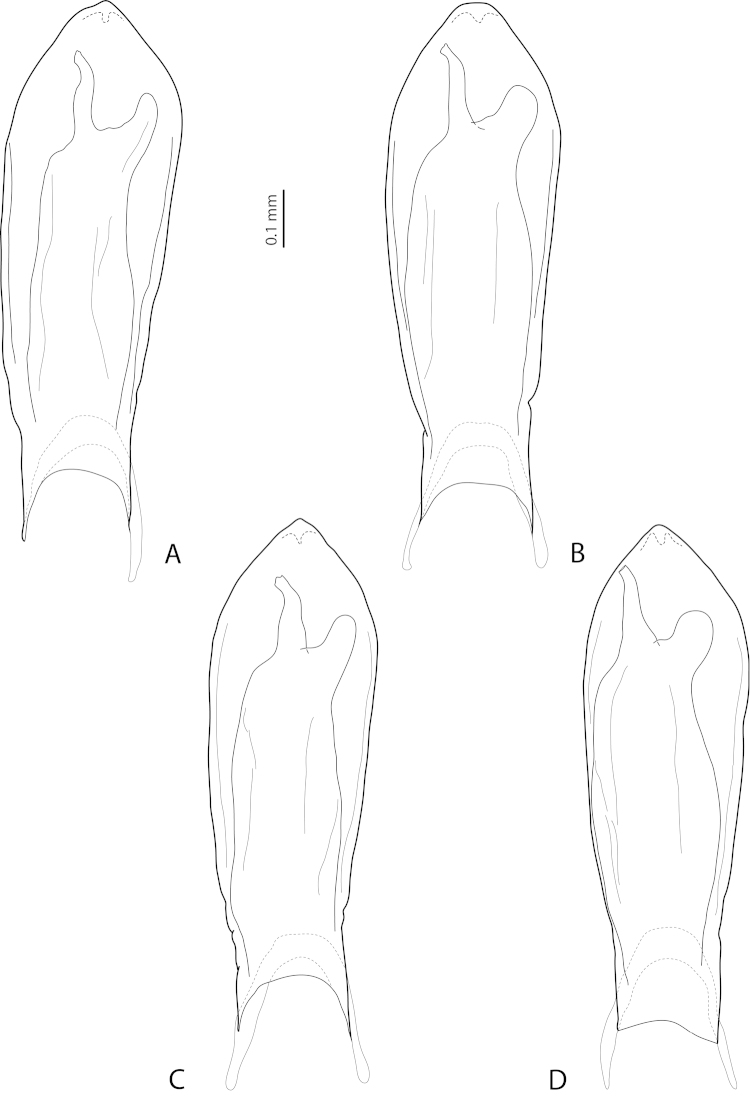
*Exocelina mondmillensis* sp. n., ventral view of median lobe. **A, B** Western Highlands, Mt. Hagen Town area, PNG 131 **C** Western Highlands, Gonzsidai-Sarup, PNG 144 **D** Enga, Anji, PNG 129. Setae not shown.

#### Distribution.

Papua New Guinea: Madang and Eastern Highlands Provinces (Fig. 14).

### 2. Exocelina hintelmannae

Taxon classificationAnimaliaColeopteraDytiscidae

(Shaverdo, Sagata & Balke, 2005)

Figs 4 , 9F, G

Papuadytes hintelmannae Shaverdo, Sagata & Balke, 2005: 272.
Exocelina hintelmannae (Shaverdo, Sagata & Balke, 2005): Nilsson 2007: 33 (comb. n.); Toussaint et al. 2014: Supplementary figs 1–4, tab. 2. (MB 1367).

#### Type locality.

Papua New Guinea: border Simbu and Eastern Highlands Provinces, Crater Mountain, between Wara Sera Station and Herowana Village, Hulene River, approximately 06°43.4'S; 145°05.6'E.

#### Type material studied.

*Holotype*: male “264 DNA M Balke” [green], “PNG Simbu / EHPr. Crater Mountain, Sera - Herowana, Wara Hulene, 1000 m, 16IX2002, Balke & Sagata (PNG 17)”, “Holotypus Papuadytes hintelmannae sp.n. des. H. Shaverdo, K. Sagata & M. Balke, 2005” (BMNH). *Paratypes*: 1 male “256 DNA M Balke” [green], “Papua New Guinea Simbu / EHPr. Crater Mountain, Wara Sera Station, 800 m, 14IX2002, Balke & Sagata (PNG 10)”, “Paratypus Papuadytes hintelmannae sp.n. des. H. Shaverdo, K. Sagata & M. Balke, 2005” (NHMW). 1 male “260 DNA M Balke” [green], “PNG Simbu / EHPr. Crater Mountain, Sera - Herowana, upper Oh River, 1200 m, 15IX2002, Balke & Sagata (PNG 12)”, “Paratypus Papuadytes hintelmannae sp.n. des. H. Shaverdo, K. Sagata & M. Balke, 2005” (NHMW).

#### Additional material.

**Simbu/EHL**: 1 female “Papua New Guinea: Simbu / EHPr. Crater Mountain, Wara Sera Station, 820m, 14IX2002, Balke & Sagata, (PNG 8)” (ZSM). 2 males, 6 females “Papua New Guinea: Simbu / EHP. Crater Mountain, Wara Sera Station, 800m, 14IX2002, Balke & Sagata, (PNG 009)” (ZSM). 17 males, 15 females “Papua New Guinea: Crater Mountain, Wara Sera Station, 800m, 14IX2002, Balke & Sagata, (PNG 010)” (NARI, NHMW, ZSM). 8 males, 9 females “Papua New Guinea Simbu/EHP. Crater Mountain, Wara Sera Station, 800m, 14IX2002, Balke & Sagata, (PNG 10)” (NHMW, ZSM). 3 males, 4 females “Papua New Guinea: Simbu / EHP. Crater Mountain, Sera - Herowana, Wara Pima, 900m, 15IX2002, Balke & Sagata (PNG 011)” (ZSM). 7 males, 14 females “Papua New Guinea: Crater Mountain, Sera - Herowana, upper Oh river, 1200m, 15IX2002, Balke & Sagata (PNG 012)” (NHMW, ZSM). 1 male, 1 female “Papua New Guinea: Crater Mountain, Sera - Herowana, Jau river, 1100m, 15IX2002, Balke & Sagata (PNG 013)” (ZSM). 8 males, 7 females “Papua New Guinea: Crater Mountain, Sera - Herowana, Jau river, 1000m, 15IX2002, Balke & Sagata (PNG 015)” (NHMW, ZSM). 12 males, 10 females “Papua New Guinea: Simbu/EHP. Crater Mountain, Sera - Herowana, Hulene river, 1000m, 16IX2002, Balke & Sagata (PNG 017)” (NHMW, ZSM). 25 males, 33 females “Papua New Guinea: Simbu / EHP. Crater Mountain, Herowana, Yawasa River, 1200m, 17.IX.2003, Balke & Sagata (PNG 019)” (NARI, NHMW, ZSM). **Simbu**: 8 males, 13 females “Papua New Guinea: Supa Haia, 1032, 10.xi.2002, K. Sagata, (WB1)” (NHMW, ZSM). **EHL**: 1 male “Stn. No. 177a”, “NEW GUINEA: E. Highland Dist., Wanatabe Valley. Nr. Okapa, c. 5000 ft. 5.ii.1965”, “M.E. Bacchus. B.M. 195-120” (BMNH). **Gulf**: 30 males, 30 females “Papua New Guinea: Gulf, Marawaka, nr. Ande, 1000m, 10.xi.2006, 07.03.598S 145.4.375E, Balke & Kinibel (PNG 89)”, one of them an additional green label “DNA M.Balke 1367” (NHMW, ZSM). 32 males, 35 females “Papua New Guinea: Gulf Province, Marawaka, Mala, 1400m, 11.xi.2006, 07.05.664S 145.44.467E, Balke & Kinibel, (PNG 90)” (NARI, NHMW, ZSM). 16 male, 12 females “Papua New Guinea: Gulf, Marawaka, Andakombe towards Morobe, 1100m, 12.xi.200, 07.09.766S 145.46.333E, Balke & Kinibel (PNG 92)” (NHMW, ZSM). 1 male, 3 females “Papua New Guinea: Gulf, Marawaka, Andakombe towards Morobe, 1500m, 12.xi.200, 07.10.413S 145.49.555E, Balke & Kinibel (PNG 93)”, the male with an additional green label “DNA M.Balke 1362” (ZSM).

#### Additions to the description

(original description in Shaverdo et al. 2005, p. 272). *Size and shape*: Beetles medium-sized (TL-H 3.4–4.15 mm, TL 3.75–4.55 mm, MW 1.8–2.2 mm; holotype: TL-H 3.9 mm, TL 4.3 mm, MW 2.05 mm), see Fig. 4.

*Female*: Without evident differences in external morphology from males, except for not modified protarsi and abdominal ventrite 6 without striae.

*Variability*: Beetles vary in shape of the median lobe and its ventral sclerite: mainly shape and length of its left lobe (Fig. 9F, G).

#### Distribution.

Papua New Guinea: Simbu, Eastern Highlands, and Gulf Provinces (Fig. 14).

### 3. Exocelina marinae

Taxon classificationAnimaliaColeopteraDytiscidae

(Shaverdo, Sagata & Balke, 2005)

Figs 5 , 10

Papuadytes marinae Shaverdo, Sagata & Balke 2005: 272.
Exocelina marinae (Shaverdo, Sagata & Balke, 2005): Nilsson 2007: 34 (comb. n.); Toussaint et al. 2014: Supplementary figs 1–4, tab. 2 (Exocelina marinae MB1291).

#### Type locality.

Papua New Guinea: Sandaun Province, trail from Telefomin to Eliptamin.

#### Type material studied.

*Holotype*: male “Papua N. G.: Sandaun Prov. Telefomin, 16.–17.5.1998 trail to Eliptamin 1700-1800 m; leg. Riedel”, “Holotypus Papuadytes marinae sp.n. des. H. Shaverdo, K. Sagata & M. Balke, 2005” (NHMW).

#### Additional material.

**Sandaun**: 4 males, 6 females “Papua New Guinea: Sandaun, Mianmin, 670m 20.x.2008, 4.53.292S 141.34.118E, Ibalim (PNG 191)”, two males with additional green labels “DNA M.Balke 3733” and “DNA M.Balke 3734” (NHMW, ZSM). 2 females “Papua New Guinea: Sandaun, Mianmin (river), 990m, 23.x.2008, 4.54.570S 141.35.490E, Ibalim (PNG 192)”, one of them with an additional green label “DNA M.Balke 3737” (ZSM). 1 male, 1 female “Papua New Guinea: Sandaun, Mianmin (pool), 990m, 23.x.2008, 4.54.570S 141.35.490E, Ibalim (PNG 193)”, the female with an additional green label “M.Balke 3777 DNA” (ZSM). 2 males, 2 females “Papua New Guinea: Sandaun, Mianmin (river), 1080m, 24.x.2008, 04.55.780S 141.38.185E, Ibalim (PNG 195)”, one male and one female with additional green labels “DNA M.Balke 3742” and “DNA M.Balke 3741”, respectively (NHMW, ZSM). 1 male, 5 females “Papua New Guinea: Sandaun, Mianmin (pool), 1080m, 24.x.2008, 04.55.780S 141.38.185E, Ibalim (PNG 196)” (NHMW, ZSM). 1 female “Papua New Guinea: Sandaun, Mianmin (river), 700m, 21.x.2008, 04.52.858S 141.31.706E, Ibalim (PNG 197)” (ZSM). 1 female “Papua New Guinea: Sandaun, Mianmin area, >1000m, 26.xii.2009, Ibalim & Pius (PNG233)” (ZSM). 1 male, 5 females “Papua New Guinea: Sandaun, Mianmin area, >1000m, 23.12.2009, Ibalim & Pius (PNG234)”, the male with an additional label “DNA M.Balke 4934” (NHMW, ZSM). 8 males, 7 females “Papua New Guinea: Sandaun, Mianmin area, >600m, 13.i.2010, Ibalim & Pius (PNG235)”, one male and one female with additional labels “DNA M.Balke 4938” and “DNA M.Balke 4931”, respectively (NHMW, ZSM). 1 male “Papua New Guinea: Sandaun, Mianmin area, >600m, 13.i.2010, Ibalim & Pius (PNG236)” (ZSM). 1 male, 1 female “Papua New Guinea: Sandaun, Mianmin area, >700m, 14.i.2010, 0 4 54.540S 141 36.953E, Ibalim & Pius (PNG238)”, the female with an additional label “DNA M.Balke 4933” (ZSM).

**Hela**: 1 male “Papua New Guinea: Southern Highlands, Tari to Koroba, 1600m, 15.v.2006, 05.46.500S 142.50.000E, Balke (PNG 65)”, “DNA M.Balke 1291” [green] (ZSM).

#### Additions to the description

(original description in Shaverdo et al. 2005, p. 270). *Male*: Median lobe with apex slightly curved in lateral view and more or less rounded in ventral view. Its ventral sclerite with unequal apical lobes: left lobe very long (very often with broken apex) and right lobe short, relatively narrow (Fig. 10).

*Female*: Without evident differences in external morphology from males, except for not modified protarsi and abdominal ventrite 6 without striae.

*Variability*: Beetles small to medium-sized: for Mianmin populations: TL-H 3.3–3.55 mm, TL 3.6–3.95 mm, MW 1.75–1.95 mm; for the holotype (Telefomin): TL-H 3.6 mm, TL 4.1 mm, MW 1.9 mm (Fig. 5); for the specimen from Tari-Koroba: TL-H 3.85 mm, TL 4.25 mm, MW 2.15 mm. Also beetles vary in shape of the median lobe and its ventral sclerite as shown in Fig. 10.

#### Distribution.

Papua New Guinea: Sandaun and Hela Provinces (Fig. 14). This species is mainly known from the south of Sandaun Province: Mianmin area. Based on the record from Tari-Koroba, we assume that the species occurs also further southeast.

### 4. Exocelina mondmillensis sp. n.

Taxon classificationAnimaliaColeopteraDytiscidae

http://zoobank.org/810826A5-0AA1-47E6-9096-24D7EFC09805

Figs 6 , 11 , 12

#### Type locality.

Papua New Guinea: Western Highlands Province, 5 km SE from Minj, Mondmill, 05°56.80'S, 144°39.90'E.

#### Type material.

*Holotype*: male “Papua New Guinea: Western Highlands, Mondmill, 5 Km SE Minj, small pools near creek, 1741 m, 12.vi.2006, 05.56.801S 144.39.898E, John (PNG 77)” (ZSM). *Paratypes*: **Western Highlands**: 53 males, 80 females with the same labels as the holotype (NARI, NHMW, ZSM). 81 males “Papua New Guinea: Western Highlands, Kurumul, 6 Km SW Kudjip, small stream, 1580 m, 13.vi.2006, 05.53.426S 144.36.600E, John (PNG 78)”, one of them with an additional green label “DNA M.Balke 1339” (NARI, NHMW, ZSM). 69 males “Papua New Guinea: Western Highlands, Mt. Hagen town area, 1600m, 7.xii.1994 05.49.745S 144.22.357E Balke & Kinibel (PNG 131)” (NARI, NHMW, ZSM). 1 male, 1 female “Papua New Guinea: Western Highlands, Simbai, 1800-2000m, 26.ii.2007, 05.15.872S 144.32.717E, Kinibel (PNG 134)”, the male with an additional green label “DNA M.Balke 3308” (ZSM). 9 males, 7 females “Papua New Guinea: Western Highlands, Simbai, Ineng River, 2000m, 27.ii.2007, 05.14.943S 144.32.818E, Kinibel (PNG 135)” (ZSM). 18 males, 21 females “Papua New Guinea: Western Highlands, Simbai, 2000m, 28.ii.2007, 05.15.174S 144.32.812E, Kinibel (PNG 136)” (NHMW, ZSM). 6 males, 1 female “Papua New Guinea: Western Highlands, Simbai, Fundum, 2000m, 1.iii.2007, 05.15.03S 144.30.867E, Kinibel (PNG 137)” (NHMW, ZSM). 23 males, 11 females “Papua New Guinea: Western Highlands, Simbai, 1800-2000m, 1.iii.2007, 05.14.276S 144.28.741E, Kinibel (PNG 138)” (NHMW, ZSM). 53 males “Papua New Guinea: Western Highlands, Simbai, Kairong River, 1850m, 2.iii.2007, 05.14.840S 144.28.457E, Kinibel (PNG 139)” (NHMW, ZSM). 4 males “Papua New Guinea: Western Highlands, Simbai - Jimi, 1500m, 2.iii.2007, 05.16.074S 144.27.886E, Kinibel (PNG 140)” (NHMW, ZSM). 1 male “Papua New Guinea: Western Highlands, Jimi, 1500m, 2.iii.2007, 05.16.335S 144.27.930E, Kinibel (PNG 141)” (ZSM). 18 males “Papua New Guinea: Western Highlands, Kundum, 1400m, 03.iii.2007, 05.16.096S 144.27.869E, Kinibel (PNG 142)” (NHMW, ZSM). 42 males “Papua New Guinea: Western Highlands, Lugup River, 1700m, 4.iii.2007, 05.17.237S 144.28.214E, Kinibel (PNG 143)” (NHMW, ZSM). 40 males “Papua New Guinea: Western Highlands, Gonzsidai-Sarup, 1700m, 4.iii.2007, 05.19.060S 144.28.671E, Kinibel (PNG 144)” one male with an additional green label “DNA M.Balke 3313” (NHMW, ZSM). 15 males “Papua New Guinea: Western Highlands, Above Sendiap, 1400m, 5.iii.2007, 05.19.774S 144.28.307E, Kinibel (PNG 145)” (NHMW, ZSM). 2 males “Papua New Guinea: Western Highlands, Jimi Valley, above Sendiap Station, 950m, 6.iii.2007, 05.20.587S 144.28.847E, Kinibel (PNG 147)” (ZSM). **Enga**: 17 males, 17 females “Papua New Guinea: Enga, nr Wabag, 1800m, 6.xii.2006, 05.30.124S 143.44.459E, Balke & Kinibel, (PNG 125)”, one male with an additional green label “DNA M.Balke 1525” (NHMW, ZSM). 2 males “Papua New Guinea: Enga, nr Wapanamanda, 1700m, 6.xii.2006, 05.36.541S 143.52.559E, Balke & Kinibel (PNG 127)”, one male with an additional green label “DNA M.Balke 1526” (ZSM). 8 males “Papua New Guinea: Enga, Wapanamanda, 1500m, 6.xii.2006, 05.38.105S 143.55.338E, Balke & Kinibel, (PNG 128)” (NHMW, ZSM). 12 males, 3 females “Papua New Guinea: Enga, Anji, 1900m, 6.xii.2006, 05.42.109S 143.55.635E, Balke & Kinibel, (PNG 129)” (NHMW, ZSM). **Madang**: 5 males, 14 females “Papua New Guinea: Madang, Keki, Adalbert Mts., 400m, 29.xi.2006, 04.43.058S 145.24.437E, Binatang Boys, (PNG 119)” (NHMW, ZSM).

#### Females of doubtful identity.

**Western Highlands**: 142 females “Papua New Guinea: Western Highlands, Kurumul, 6 Km SW Kudjip, small stream, 1580 m, 13.vi.2006, 05.53.426S 144.36.600E, John (PNG 78)” (NARI, NHMW, ZSM). These females are a mixture of three species: *Exocelina mondmillensis* sp. n., *Exocelina edeltraudae* (Shaverdo, Hendrich & Balke, 2012), and *Exocelina damantiensis* (Balke, 1998). 47 females “Papua New Guinea: Western Highlands, Mt. Hagen town area, 1600m, 7.xii.1994 05.49.745S 144.22.357E Balke & Kinibel (PNG 131)” (NARI, ZSM). These females are a mixture of two species: *Exocelina mondmillensis* sp. n. and *Exocelina edeltraudae*. 50 females “Papua New Guinea: Western Highlands, Simbai, Kairong River, 1850m, 2.iii.2007, 05.14.840S 144.28.457E, Kinibel (PNG 139)” (NARI, ZSM). 10 females “Papua New Guinea: Western Highlands, Simbai - Jimi, 1500m, 2.iii.2007, 05.1.074S 144.27.886E, Kinibel (PNG 140)” (ZSM). 7 females “Papua New Guinea: Western Highlands, Jimi, 1500m, 2.iii.2007, 05.16.335S 144.27.930E, Kinibel (PNG 141)” (ZSM). 33 females “Papua New Guinea: Western Highlands, Kundum, 1400m, 03.iii.2007, 05.16.096S 144.27.869E, Kinibel (PNG 142)” (ZSM). These females are a mixture of two species: *Exocelina mondmillensis* sp. n. and *Exocelina jimiensis* Shaverdo & Balke, 2014. 26 females “Papua New Guinea: Western Highlands, Gonzsidai-Sarup, 1700m, 4.iii.2007, 05.19.060S 144.28.671E, Kinibel (PNG 144)” (ZSM). These females are a mixture of two species: *Exocelina mondmillensis* sp. n. and *Exocelina edeltraudae*. 34 females “Papua New Guinea: Western Highlands, Lugup River, 1700m, 4.iii.2007, 05.17.237S 144.28.214E, Kinibel (PNG 143)” (NHMW, ZSM). 9 females “Papua New Guinea: Western Highlands, Above Sendiap, 1400m, 5.iii.2007, 05.19.774S 144.28.307E, Kinibel (PNG 145)” (ZSM). 9 females “Papua New Guinea: Western Highlands, Jimi Valley, above Sendiap Station, 950m, 6.iii.2007, 05.20.587S 144.28.847E, Kinibel (PNG 147) (ZSM). These females are a mixture of two species: *Exocelina mondmillensis* sp. n. and *Exocelina madangensis* (Balke, 2001). **Enga**: 10 females “Papua New Guinea: Enga, Wapanamanda, 1500m, 6.xii.2006, 05.38.105S 143.55.338E, Balke & Kinibel, (PNG 128)” (ZSM). These females are a mixture of two species: *Exocelina mondmillensis* sp. n. and *Exocelina madangensis* (Balke, 2001).

#### Diagnosis.

Beetle medium-sized, brown to piceous, with reddish head and pronotal sides, shiny; median lobe with slightly curved downwards apex but thin in lateral view and ventral sclerite with two unequal apices (left one long, narrow and curved and right one short, broad and more or less rounded). The species is similar to *Exocelina marinae* and *Exocelina pseudomarinae* sp. n. in shape of the ventral sclerite of median lobe, but distinctly differs from them in having fine, inconspicuous punctation and weak microreticulation on the dorsal surface and a thin tip of the median lobe. From *Exocelina broschii* and *Exocelina hintelmannae*, it can be distinguished by the shape of the median lobe (thin apex) and its ventral sclerite (very long left lobe), and from the former also by finer elytral punctation.

#### Description.

*Size and shape*: Beetle medium-sized (TL-H 3.5–4.1 mm, TL 3.9–4.5 mm, MW 1.8–2.2 mm), with oblong-oval habitus, broadest at elytral middle. *Coloration*: Head reddish brown to piceous, usually darker medially and posterior to eyes, sometimes almost uniform; pronotum with brown to piceous medially and reddish brown to brown sides; elytra brown to piceous, usually with narrow reddish sutural lines; head appendages and legs yellowish to reddish, legs distally darker, especially metathoracic legs (Fig. 6). Teneral specimen with coloration paler.

*Surface sculpture*: Head with dense punctation (spaces between punctures 1–3 times size of punctures), evidently finer and sparser anteriorly; diameter of punctures smaller than diameter of cells of microreticulation. Pronotum with much sparser and finer punctation than head. Elytra with very sparse and fine punctation, almost invisible. Pronotum and elytra with weakly impressed microreticulation, dorsal surface shiny. Head with microreticulation stronger. Metaventrite and metacoxae distinctly microreticulate, metacoxal plates with longitudinal strioles and transverse wrinkles. Abdominal ventrites with distinct microreticulation, strioles, and very fine and sparse punctation.

*Structures*: Pronotum with distinct lateral bead. Base of prosternum and neck of prosternal process with distinct ridge, slightly rounded anteriorly. Blade of prosternal process lanceolate, relatively narrow, slightly convex, with distinct lateral bead and few setae; neck and blade of prosternal process evenly jointed. Abdominal ventrite 6 broadly rounded or slightly truncate.

*Male*: Antenna simple (Fig. 6). Protarsomere 4 with large, thick, strongly curved anterolateral hook-like seta. Protarsomere 5 ventrally with anterior row of 18 and posterior row of 4 short setae (Fig. 11A). Median lobe with thin apex, slightly curved in lateral view and pointed in ventral view. Its ventral sclerite with two unequal apices (left one long, narrow and curved and right one short, broad and more or less rounded). Paramere with subdistal setae denser and thicker than proximal setae (Fig. 11B–D). Abdominal ventrite 6 with 4–7 lateral striae on each side.

*Holotype*: TL-H 4.05 mm, TL 4.45 mm, MW 2.15 mm.

*Female*: Without evident differences in external morphology from males, except for not modified protarsi and abdominal ventrite 6 without striae.

*Variability*: Beetles vary mainly in shape of the ventral sclerite of the median lobe as shown in Fig. 12.

#### Distribution.

Papua New Guinea: Western Highlands, Enga, and Madang Provinces (Fig. 14).

#### Etymology.

The name refers to Mondmill, the type locality, where the species was found in great numbers. The name is an adjective in the nominative singular.

### 5. Exocelina pseudomarinae sp. n.

Taxon classificationAnimaliaColeopteraDytiscidae

http://zoobank.org/60127776-664C-425F-962E-B40E1F9B7E74

Figs 7 , 13

Exocelina undescribed sp. MB1287: Toussaint et al. 2014: Supplementary figs 1–4, tab. 2.

#### Type locality.

Papua New Guinea: Hela Province, Tari, 05°50.38'S, 142°55.90'E.

#### Type material.

*Holotype*: male “Papua New Guinea: Southern Highlands, Tari (trickle in gardenland), 1700m, 12.v.2006, 05.50.383S 142.55.901E, Balke (PNG 58)”, “DNA M.Balke 1287” [green] (ZSM). *Paratypes*: 2 males, 1 female with the same labels as the holotype (NHMW, ZSM).

#### Diagnosis.

Beetle medium-sized, brown to dark brown, with reddish head and pronotal sides, submatt; median lobe with apex strongly curved downwards in lateral view and ventral sclerite with two unequal apices (left one long, narrow and curved apically and right one short, broad and more or less strait). The species is similar to *Exocelina marinae* Shaverdo, Sagata & Balke, 2005 from which distinctly differs in larger size, sparser and finer punctation and weaker microreticulation of the dorsal surface, and strongly curved apex of the median lobe, which is similar to that of *Exocelina hintelmannae* Shaverdo, Sagata & Balke, 2005. The specimen of *Exocelina marinae* from Tari-Koroba, though large in size and with the same distribution, has a distinctly stronger sculpture on the dorsal surface and a median lobe with only a slightly curved apex in lateral view and a narrower right lobe of the ventral sclerite. Therefore, it can be easily distinguished from *Exocelina pseudomarinae* sp. n.

#### Description.

*Size and shape*: Beetle medium-sized (TL-H 3.55–4.05 mm, TL 3.85–4.4 mm, MW 1.9–2.1 mm), with oblong-oval habitus, broadest at elytral middle. *Coloration*: Head reddish brown, brown to dark brown medially and posterior to eyes; pronotum with brown to dark brown medially (to piceous – narrow part on disc) and reddish brown sides; elytra brown to dark brown, sometimes with narrow reddish brown sutural lines; head appendages yellowish, legs yellowish to reddish, distally darker, especially metathoracic legs (Fig. 7). Teneral specimen with coloration slightly paler.

*Surface sculpture*: Head with very dense punctation (spaces between punctures 1–2 times size of punctures), evidently finer and sparser anteriorly; diameter of punctures smaller than diameter of cells of microreticulation or equal to it. Pronotum and elytra with punctation sparser and finer than on head, but very distinct. Pronotum and elytra with distinct microreticulation, dorsal surface submatt. Head with microreticulation stronger. Metaventrite and metacoxae distinctly microreticulate, metacoxal plates with longitudinal strioles and transverse wrinkles. Abdominal ventrites with distinct microreticulation, strioles, and sparse punctation, coarser on two last abdominal ventrites.

*Structures*: Pronotum with distinct lateral bead. Base of prosternum and neck of prosternal process with distinct ridge, slightly rounded anteriorly. Blade of prosternal process lanceolate, relatively narrow, slightly convex, with distinct lateral bead and few setae; neck and blade of prosternal process evenly jointed. Abdominal ventrite 6 broadly rounded or slightly truncate.

**Figure 13.**
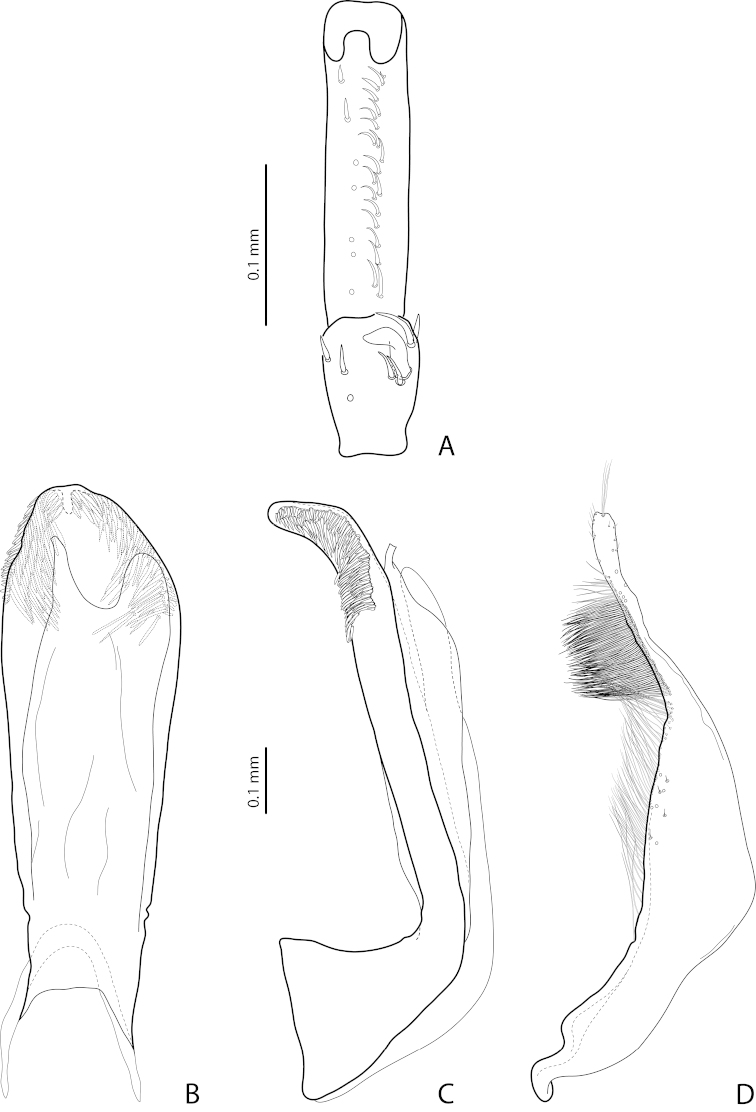
*Exocelina pseudomarinae* sp. n., Southern Highlands, Tari, PNG 58 **A** male protarsomeres 4–5 in ventral view **B** median lobe in ventral view **C** median lobe in lateral view **D** paramere in external view.

*Male*: Antenna simple (Fig. 7). Protarsomere 4 with large, thick, strongly curved anterolateral hook-like seta. Protarsomere 5 ventrally with anterior row of 20 and posterior row of 7 short setae (Fig. 13A). Median lobe with strongly curved apex in lateral view and more or less rounded in ventral view. Its ventral sclerite with two unequal apices (left one long, narrow and curved apically and right one short, broad and more or less strait). Paramere with subdistal setae denser and thicker than proximal setae (Fig. 13B–D). Abdominal ventrite 6 with 6–8 lateral striae on each side.

*Holotype*: TL-H 4.05 mm, TL 4.4 mm, MW 2.1 mm.

*Female*: Without evident differences in external morphology from males, except for not modified protarsi and abdominal ventrite 6 without striae.

#### Distribution.

**Figure 14.**
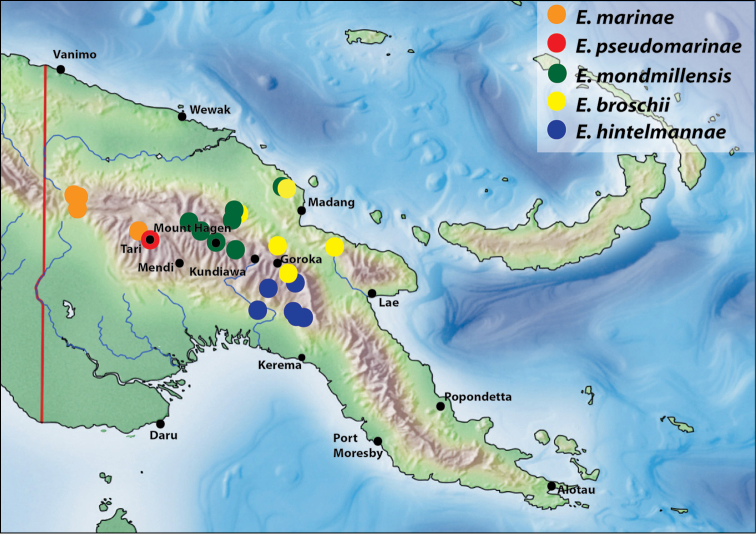
Map of Papua New Guinea showing distribution of species of the *Exocelina broschii*-group.

Papua New Guinea: Hela Province. This species is known only from the type locality (Fig. 14).

#### Etymology.

The name points to similarity of the new species to *Exocelina marinae*. The name is a noun in the nominative singular standing in apposition.

### Key to species of the *Exocelina broschii*-group

The key is based mostly on male characters. In many cases females cannot be assigned to a species due to the similarity of their external and internal structures (for female genitalia see Figs 17a and 17b in Shaverdo et al. (2005)). Some species are rather similar in external morphology, therefore, in most cases, the male genitalia need to be studied for reliable species identification. Numbers in parentheses refer to the order of species descriptions given above.

**Table:** 

1	Dorsal surface of the body matt, with strongly impressed microreticulation and dense coarse punctation or submatt, with finer microreticulation and punctation (Figs 5, 7). Median lobe with apex broad, slightly or strongly curved in lateral view and ventral sclerite with two apical lobes of different length and shape: left one long (it can be broken apically) and narrow and right one shorter and broader (Figs 10, 13)	**2**
–	Dorsal surface of the body shiny, with distinctly weaker microreticulation and finer punctation (Figs 1–4, 6). Median lobe with apex thin, slightly curved in lateral view and ventral sclerite with two apical lobes of different length and shape (Figs 11B, 12) or with apex broad, slightly or strongly curved in lateral view and ventral sclerite with two apical lobes of more or less equal length and shape	**3**
2	Dorsal surface of body matt, with strongly impressed microreticulation and dense coarse punctation (Fig. 5); apex of median lobe slightly curved in lateral view (Fig. 10D–F, fig. 12a in Shaverdo et al. (2005))	(3) ***marinae***
–	Dorsal surface of body submatt, with punctation evidently sparser and finer and microreticulation weaker (Fig. 7); apex of median lobe more strongly curved in lateral view (Fig. 13C)	(5) ***pseudomarinae* sp. n.**
3	Dorsal surface with very fine and sparse to moderately fine and dense punctation (Figs 1–3); median lobe with apex broad, slightly curved in lateral view and ventral sclerite with two more or less equal apical lobes (Figs 8, 9A–E)	(1) ***broschii***
–	Dorsal surface with very fine and sparse punctation (Figs 4, 6); shape of median lobe or its ventral sclerite different	**4**
4	Median lobe with apex broad, strongly curved in lateral view, its ventral sclerite with two more or less equal tips (Fig. 9A–E, figs 3,13a in Shaverdo et al. (2005))	(2) ***hintelmannae***
–	Media lobe with apex thin, slightly curved in lateral view, its ventral sclerite with two tips of different length: left one very long and narrow and right one shorter and broader, somehow rounded (Figs 11, 12)	(4) ***mondmillensis* sp. n.**

## Supplementary Material

XML Treatment for Exocelina broschii

XML Treatment for Exocelina hintelmannae

XML Treatment for Exocelina marinae

XML Treatment for Exocelina mondmillensis

XML Treatment for Exocelina pseudomarinae
